# Complex Lymphatic Anomaly Presenting with Chylothorax, Chylous Ascites, and Generalized Subcutaneous Edema in a Young Cat: Comparative Insights Based on the Human ISSVA Classification

**DOI:** 10.3390/vetsci12121199

**Published:** 2025-12-15

**Authors:** Kazuyuki Terai, Aki Takeuchi, Ikki Mitsui, Tomohiro Yoshida, Akari Hatanaka, Ahmed Farag, Ryou Tanaka

**Affiliations:** 1 Department of Veterinary Medicine, Faculty of Agriculture, Tokyo University of Agriculture and Technology, Fuchu 183-8509, Japan; fq9913@go.tuat.ac.jp (K.T.); fv5028@go.tuat.ac.jp (A.T.); fy3391@go.tuat.ac.jp (T.Y.); s257982z@st.go.tuat.ac.jp (A.H.); s210594v@st.go.tuat.ac.jp (A.F.); 2 No Boundaries Animal Pathology, Fuchu 183-0051, Japan; mitsui@no-boundaries.jp; 3 Department of Surgery, Anesthesiology, and Radiology, Faculty of Veterinary Medicine, Zagazig University, Zagazig 44519, Egypt

**Keywords:** complex lymphatic anomaly, generalized lymphatic anomaly, central conducting lymphatic anomaly, lymphangiomatosis, chylothorax, chylous ascites, cat, lymphangiography, comparative pathology, ISSVA classification

## Abstract

In human medicine, the 2025 International Society for the Study of Vascular Anomalies (ISSVA) classification distinguishes localized lymphatic malformations from systemic disorders referred to as complex lymphatic anomalies (CLAs), including generalized lymphatic anomaly (GLA) and central conducting lymphatic anomaly (CCLA). GLA is characterized by multifocal proliferation of dilated lymphatic channels infiltrating soft tissues and visceral organs, whereas CCLA involves structural or functional abnormalities of the central lymphatic pathways such as the thoracic duct commonly resulting in chylous effusion. Reports of systemic lymphatic disease in veterinary species are exceedingly rare. A five-month-old intact male domestic shorthair cat presented with progressive dyspnea and was diagnosed with chylothorax and chylous ascites. Computed tomography (CT) revealed contrast leakage from both the thoracic duct and aortic hiatus, along with diffuse subcutaneous edema, indicating a systemic lymphatic disorder. Histopathology demonstrated proliferative lymphatic channels within the subcutaneous tissues, consistent with GLA, while imaging findings suggested concurrent CCLA, supporting classification as an overlapping CLA phenotype. Despite medical and surgical interventions, the cat developed progressive edema and recurrent pleural effusion and ultimately died. This case demonstrates that cats can develop systemic lymphatic disorders analogous to human CLAs and highlights the diagnostic value of CT lymphangiography. Furthermore, it underscores the utility of applying the ISSVA classification to enhance comparative understanding of lymphatic diseases in veterinary medicine.

## Simple Summary

The lymphatic system plays an essential role in transporting fluid, dietary fats, and immune cells throughout the body. When this system develops abnormally, it can lead to the accumulation of lymphatic fluid within the chest or abdominal cavity, as well as widespread swelling beneath the skin. In human medicine, lymphatic disorders are categorized based on the specific regions of the lymphatic system that are affected. Comparable conditions are uncommon in animals. This report describes a young cat presenting with severe respiratory distress caused by accumulation of fluid in the chest and abdomen, along with generalized subcutaneous swelling. Imaging identified lymphatic leakage from two regions of the thoracic duct, and histopathology confirmed abnormal proliferation of small lymphatic vessels. The clinical and pathological findings were consistent with a complex lymphatic anomaly, a condition in humans, characterized by structural abnormalities of lymphatic vessels and impaired lymph flow. Despite therapeutic intervention, the cat did not survive. This case contributes to a better understanding of lymphatic diseases in veterinary medicine and offers a comparative perspective relevant to human clinical practice.

## 1. Introduction

The lymphatic system plays a critical role in maintaining tissue fluid homeostasis, facilitating lipid absorption, and supporting immune cell transport [1]. Developmental or functional abnormalities within this system give rise to a heterogeneous group of disorders collectively referred to as lymphatic malformations [2,3,4,5,6]. In human medicine, the updated International Society for the Study of Vascular Anomalies (ISSVA) 2025 classification categorizes lymphatic malformations as slow-flow vascular malformations, subdividing them into isolated lymphatic malformations, representing localized structural abnormalities, and complex lymphatic anomalies (CLAs), which reflect systemic involvement [2]. CLAs encompass generalized lymphatic anomaly (GLA), central conducting lymphatic anomaly (CCLA), Gorham–Stout disease (GSD), and kaposiform lymphangiomatosis (KLA) [2]. Within this framework, GLA is synonymous with lymphangiomatosis, with the updated terminology emphasizing its developmental rather than neoplastic origin [2]. GLA is characterized by multifocal proliferation and dilation of lymphatic channels infiltrating soft tissues and visceral organs. In contrast, CCLA involves structural or functional abnormalities of central lymphatic conduits, such as the thoracic duct, and is frequently associated with chylothorax or chylous ascites [3].

In veterinary medicine, systemic lymphatic disorders are exceedingly rare and remain poorly characterized. Most previously reported cases have been described as localized lymphangioma or lymphangiomatosis, terminology historically used in veterinary reports prior to the adoption of the ISSVA classification (2025) [7,8,9,10,11,12,13]. Although a single feline case of mesenteric lymphatic malformation associated with chylothorax has been documented, to the best of our knowledge, no previous report has described a cat presenting with simultaneous involvement of the thoracic, abdominal, and subcutaneous lymphatic systems.

Here, we describe a feline case of systemic lymphatic abnormality characterized by chylothorax, chylous ascites, and generalized subcutaneous edema. When interpreted through the lens of the human ISSVA framework, this condition most closely resembles GLA or CCLA. We present clinical, imaging, surgical, and histopathological findings, and discuss the diagnostic approach and classification in comparison with established concepts in human medicine.

## 2. Case Presentation

### 2.1. Signalment and Clinical History

A five-month-old intact male domestic shorthair cat was referred to our hospital for evaluation of progressive tachypnea. The referring veterinarian had identified pleural effusion on thoracic radiographs. Thoracocentesis yielded 120 mL of milky fluid, and biochemical analysis showed triglyceride concentration >375.0 mg/dL, exceeding the serum triglyceride concentration (62.0 mg/dL). Based on these findings, chylothorax was diagnosed. Repeated thoracocentesis every three days failed to reduce the effusion volume, and the cat was referred for further investigation and treatment.

At presentation, the owner reported a marked decline in activity (approximately 10% of normal) and a reduction in appetite to approximately 20%. Occasional facial swelling had also been noted. There was no history of trauma or previous surgery. The body weight was 2.6 kg, and the cat appeared mildly emaciated (body condition score 2/5). Physical examination revealed a body temperature of 38.6 °C, heart rate of 180 beats/min, and respiratory rate of 56 breaths/min, consistent with tachypnea.

### 2.2. Diagnostic Evaluation

Hematologic analysis (IDEXX ProCyte Dx; IDEXX Laboratories, Inc., Westbrook, ME, USA) and serum biochemistry (DRI-CHEM NX700; FUJIFILM Corporation, Tokyo, Japan) were performed at the initial presentation. The results, including reference intervals, are summarized in Table 1. Tests for feline immunodeficiency virus (FIV) antibody and feline leukemia virus (FeLV) antigen were negative.

Thoracic radiographs demonstrated increased soft-tissue opacity within the lung fields, clear visualization of interlobar fissures, and blurring of the ventral cardiac silhouette, consistent with pleural effusion. Abdominal ultrasonography revealed a small anechoic region surrounding the urinary bladder, indicative of mild ascites. Ultrasound-guided thoracentesis and abdominocentesis were performed for cytologic and biochemical evaluation.

**Figure 1:**
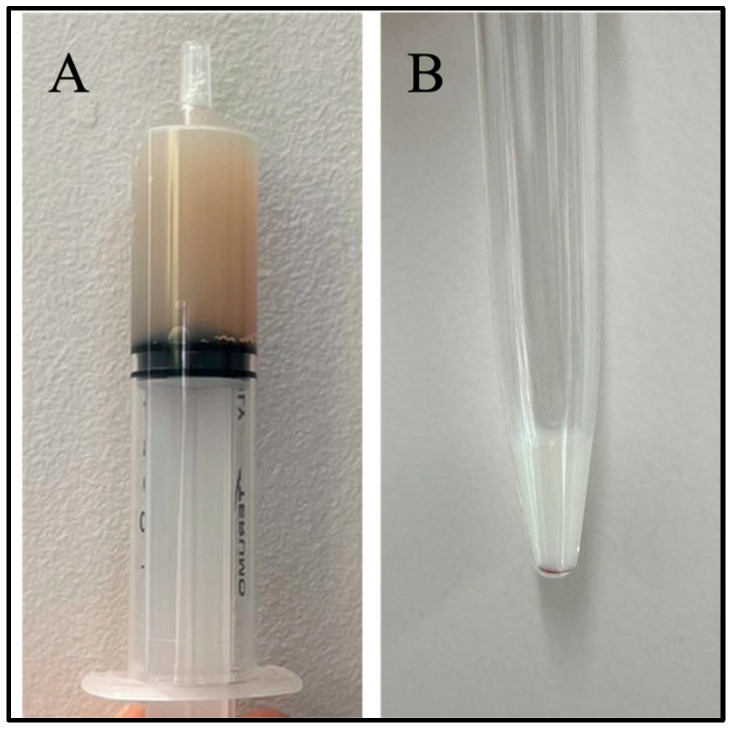
Macroscopic appearance of the effusions. (**A**) The pleural fluid appeared turbid and milky white, consistent with chylous effusion. Biochemical analysis revealed a triglyceride concentration exceeding 500 mg/dL and total protein of 4.0 g/dL, with cytology showing predominantly small lymphocytes and mild numbers of neutrophils and erythrocytes. (**B**) The ascitic fluid was clear and milky white, with a similar appearance to the pleural effusion. Triglyceride concentration exceeded 500 mg/dL, and total protein was 1.4 g/dL. Cytologic examination revealed mainly small lymphocytes without atypical or infectious cells, confirming chylous ascites.

The pleural fluid was turbid and milky white (Figure 1A). Cytology revealed predominantly small lymphocytes with mild numbers of neutrophils and erythrocytes, without atypical cells or infectious organisms. The ascitic fluid was clear and milky white (Figure 1B), and cytological findings were similar to those of the pleural fluid, with small lymphocytes predominating and no evidence of neoplasia or infection. The biochemical and physical properties of both effusions are summarized in Table 2. Echocardiography revealed no structural cardiac abnormalities.

**Figure 2:**
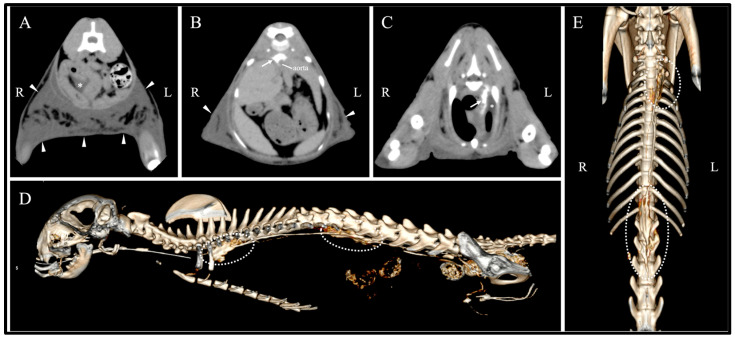
Computed tomography (CT) and CT lymphangiography findings. (**A**) Non-contrast CT image showing diffuse subcutaneous edema in the inguinal region (arrowheads) and mild ascites within the abdominal cavity (asterisk). (**B**) CT lymphangiography image revealing contrast leakage (arrow) from the thoracic duct at the aortic hiatus and subcutaneous edema (arrowheads). (**C**) CT lymphangiography image showing contrast leakage (arrow) from the thoracic duct in the cranial thoracic region near the third rib level and pleural effusion (asterisk). (**D**) Three-dimensional reconstruction (left lateral view) demonstrating contrast leakage (dotted outlines) at both the aortic hiatus and cranial thoracic region. (**E**) Three-dimensional reconstruction (ventral view) demonstrating contrast leakage (dotted outlines) at both the aortic hiatus and cranial thoracic region.

Given the presence of both chylothorax and chylous ascites and the absence of primary cardiac disease, whole-body computed tomography (CT) was performed under general anesthesia for further evaluation, including non-contrast, lymphangiographic, and angiographic phases. Non-contrast CT revealed diffuse subcutaneous edema extending from the cervical to femoral regions, most prominent in the inguinal area (average attenuation 9.6 Hounsfield units (HU)) (Figure 2A). Peritoneal effusion was present within the abdominal cavity. For lymphangiography, contrast medium (Iopamiron; Bayer Yakuhin, Ltd., Osaka, Japan) was injected into both plantar pads at 0.5 mL/kg each, followed by gentle massage for 3 min. CT images revealed contrast leakage at two sites: around the aortic hiatus and in the left anterior thoracic region near the third rib level, indicating thoracic duct leakage (Figure 2B–E). Leakage at the aortic hiatus extended across the thoracic and abdominal cavities. CT angiography revealed no mass lesions or thrombi that could account for secondary chylothorax.

### 2.3. Therapeutic Management

Given persistent chylous effusion and respiratory distress, medical therapy was initiated with rutin (100 mg/kg PO twice daily; Solaray Rutin 500 mg VegCaps 90; Solaray, Ogden, UT, USA) and a low-fat diet (Royal Canin Satiety Support Dry (Cat); Royal Canin Japon LLC, Tokyo, Japan). Despite medical management, pleural effusion did not improve, requiring daily drainage of approximately 160 mL to maintain comfort. Therefore, surgical intervention was scheduled on day 9 of hospitalization. Given the locations of lymphatic leakage, conventional thoracic duct ligation at the 10th intercostal space was unlikely to be effective. Thus, ligation was attempted near the aortic hiatus, immediately cranial or caudal to the suspected leakage site.

To enhance intraoperative visualization of lymphatic flow, a small amount of butter was given orally preoperatively. Premedication included atropine sulfate (0.05 mg/kg SC; Atropine Sulfate Injection 0.5 mg; Mitsubishi Tanabe Pharma Co., Osaka, Japan), midazolam (0.2 mg/kg IV; Dormicum injection 10 mg; Maruishi Pharmaceutical Co., Ltd., Osaka, Japan), and fentanyl (5 µg/kg IV; Fentanyl Injection 0.25 mg; Daiichi Sankyo Co., Ltd., Tokyo, Japan). Cefazolin (20 mg/kg IV; Cefazolin Sodium injection 1 mg; Nichi-Iko Pharmaceutical Co., Ltd., Toyama, Japan) was administered as prophylactic antibiotic. Anesthesia was induced with propofol (4 mg/kg IV; Propofol intravenous injection 1%; Fresenius Kabi, Japan) and maintained with isoflurane (Isoflurane for animal use; MSD Animal Health, Madison, NJ, USA) in oxygen. Continuous fentanyl infusion (10 µg/kg/h) was used for intraoperative analgesia.

The cat was positioned in left lateral recumbency, and a right 12th-intercostal thoracotomy was performed. The diaphragm was retracted caudally to expose the aortic hiatus. Upon incision of the mediastinal pleura, milky chyle began to ooze from within; however, the precise leakage site could not be identified, and the thoracic duct could not be visualized clearly. To assist localization, indocyanine green (ICG) (Diagnogreen for Injection 25 mg; Daiichi Sankyo Co., Ltd., Tokyo, Japan) was injected into both plantar pads (1.5 mL each). Green-stained chyle was observed in the mediastinum, confirming active lymphatic leakage but not the specific source. The diaphragm was incised to observe the abdominal aspect of the hiatus; however, the thoracic duct was still not detected. Because of the risk of duct injury, ligation was deemed unsafe. Instead, hemostatic material (Avitene™ (Flour type) Microfibrillar Collagen Hemostat; Becton, Dickinson and Company, Warwick, RI, USA) was applied around the aortic hiatus to induce local adhesion and fibrosis. The diaphragm was closed with absorbable sutures, a chest drain was placed, and routine thoracotomy closure was performed. Additionally, multiple punch biopsies were obtained from the skin and subcutaneous fascia of the edematous inguinal region (identified on CT) for histopathologic analysis. Immediately after the biopsy, the specimen was fixed in 10% neutral buffered formalin. After two days of fixation, the tissue was embedded in paraffin wax. For histopathological examination, 4-μm-thick sections were stained with hematoxylin and eosin (HE). Histopathologic examination of the inguinal biopsy revealed subcutaneous edema and nonsuppurative inflammation in adipose tissue, without evidence of neoplasia or infection.

### 2.4. Clinical Course and Outcome

**Figure 3:**
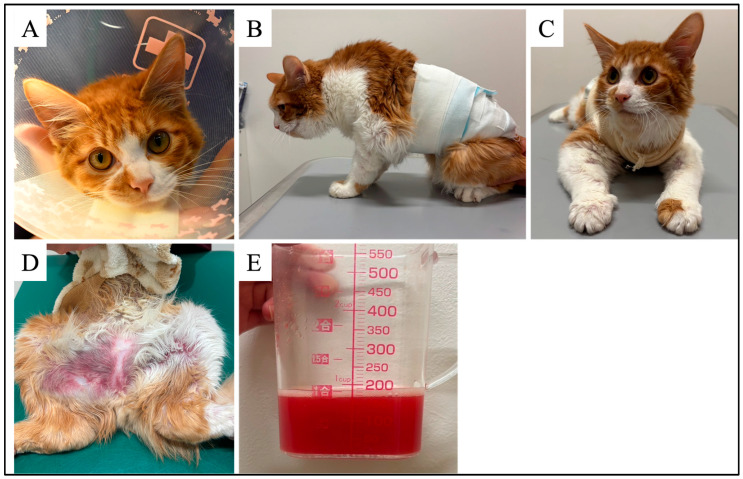
Clinical progression of edema and recurrence of pleural effusion. (**A**) Facial appearance at the initial presentation showing no apparent edema. (**B**) Left lateral view on day 24 demonstrating evident swelling extending from the mandibular to the forelimb region. (**C**) Frontal view on day 24 showing marked edema extending to the distal forelimbs. (**D**) Inguinal region on day 49 showing severe purpura corresponding to areas previously affected by edema. (**E**) Gross appearance of pleural fluid collected on day 49, showing hemorrhagic effusion.

Recovery was uneventful, and the cat was discharged on day 11. By day 24, general condition had improved, appetite was fully restored, and average daily pleural drainage volume decreased to 38 mL/day, compared with preoperative 160 mL/day. Pleural triglyceride concentration had decreased to 290 mg/dL. However, compared with the facial appearance at the initial presentation (Figure 3A), the face had become clearly edematous, and marked swelling was also evident in the cervical and forelimb regions (Figure 3B,C). Prednisolone (1 mg/kg PO daily; Prednisolone Tablets 2.5 mg; Nipro Corporation, Osaka, Japan) was initiated. By day 34, the daily drainage volume had further decreased to 7 mL/day, but erythema developed over the edematous skin, and the owner reported mild discomfort. Tramadol (2 mg/kg PO twice daily; Tramal OD Tablet 25 mg; Nippon Shinyaku Co., Ltd., Kyoto, Japan) was prescribed, resulting in symptomatic relief.

On day 49, the cat’s condition deteriorated, with tachypnea and lethargy. Severe purpura developed in areas corresponding to the regions of edema (Figure 3D). Reaccumulation of pleural effusion was confirmed, and 170 mL of hemorrhagic fluid was drained (Figure 3E). The cat’s condition continued to decline, and death occurred on day 51.

### 2.5. Postmortem Findings

**Figure 4:**
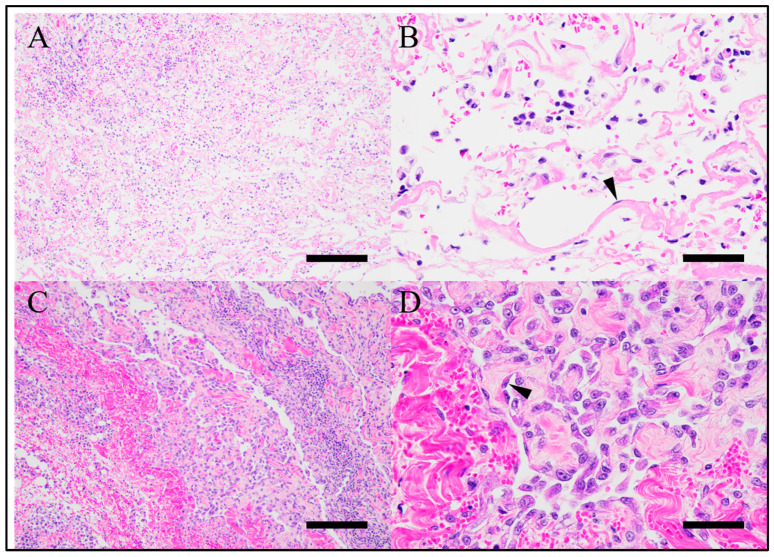
Postmortem histopathology of the skin and subcutaneous tissue. H&E stain. (**A**) Tissue of the right thigh. There are numerous anastomosing slit-like lymphatic vessels in the subcutis. Bar = 200 µm. (**B**) Tissue of the right thigh. Anastomosing slit-like lymphatic vessels are lined by bland endothelial cells (arrowhead). Interstitial edema, hemorrhage, and mild-to-severe non-suppurative inflammatory infiltrates are also noted. Bar = 50 µm. (**C**) Tissue of the right forelimb. There are numerous anastomosing slit-like lymphatic vessels in the subcutis with abundant interstitial collagen. Bar = 200 µm. (**D**) Tissue of the right forelimb. Endothelial cells lining the lymphatic vessels exhibited pleomorphism and atypia (arrowhead) indicative of malignant transformation. Bar = 50 µm.

Postmortem histopathology of the skin and subcutaneous tissue from the mandible, right forelimb, and right thigh revealed numerous anastomosing slit-like lymphatic vessels (Figure 4A,B) containing few erythrocytes, accompanied by edema, hemorrhage, and mild-to-severe inflammatory cell infiltration (macrophages, lymphocytes, plasma cells). In the right forelimb tissue, endothelial cells lining the lymphatic vessels exhibited pleomorphism and atypia, with one mitotic figure per 2.37 mm^2^ (Figure 4C,D). These findings were consistent with GLA with partial malignant transformation.

## 3. Discussion

In this young cat, chylothorax, chylous ascites, and diffuse subcutaneous edema were observed. Given the cat’s age, these findings were considered to originate from a congenital lymphatic abnormality. Because the lesions were systemic rather than localized, the condition was classified as a CLAs according to the ISSVA classification (2025) [2]. Histopathology revealed proliferative lymphatic channels within the subcutaneous tissues, consistent with GLA—historically termed lymphangiomatosis [3,14]. Some lymphatic vessels displayed atypical or malignant features, which may not represent primary lymphangiosarcoma but rather a secondary malignant transformation related to chronic lymphatic stasis, resembling Stewart–Treves syndrome [15]. Imaging also demonstrated contrast leakage from the thoracic duct, indicating a central lymphatic structural or functional defect characteristic of CCLA. Thus, this case exhibited features of both GLA and CCLA. Similar overlap between these entities has also been reported in humans, and strict separation into a single diagnostic category is often considered challenging [3].

CT lymphangiography proved invaluable in identifying multiple chyle leakage sites. In human CLAs management, lymphoscintigraphy, CT lymphangiography, and MR lymphangiography enable comprehensive structural and functional evaluation of the lymphatic system, facilitating the identification of both the extent of disease and precise localization of leakage [4,5]. Although scintigraphy and MR lymphangiography are not yet widely implemented in veterinary practice, CT-based lymphangiography represents a practical and readily applicable alternative, offering high diagnostic utility and straightforward technique [16,17]. The widespread leakage pattern observed in this cat supports the presence of diffuse structural or functional lymphatic abnormalities, rather than a single focal defect. Following CT lymphangiography, angiographic evaluation is also important to exclude secondary causes of chylothorax, such as thoracic masses or thrombosis.

At the initial stage of this case, chylothorax was the major factor impairing the cat’s quality of life, and therefore, therapeutic efforts were directed toward reducing the pleural effusion. Conventional medical management including low-fat diet, rutin supplementation, and surgical options such as thoracic duct ligation, cisterna chyli ablation, or pericardiectomy [18,19]. In this case, medical treatment was ineffective, prompting surgical intervention. CT imaging revealed leakage from the thoracic duct at both the cranial thoracic region and the aortic hiatus; based on lymphatic flow direction, it was determined that controlling the leakage at the caudal aortic hiatus would be essential. The hiatus was explored with the intent to ligate the thoracic duct if identified; however, visualization proved difficult, and an alternative approach aimed at promoting adhesion and closure of the leakage site was performed instead. This strategy resulted in temporary improvement until day 49, when hemorrhagic pleural effusion developed. Preoperative butter feeding and ICG lymphatic injections were used to visualize the duct, but the thoracic duct remained indistinct intraoperatively. Intraoperative near-infrared fluorescence lymphangiography (NIRFL) using ICG could potentially improve duct identification [20], though it was not available at our facility.

Progressive generalized subcutaneous edema also caused discomfort, which was partially alleviated with tramadol; however, prednisolone at anti-inflammatory doses failed to achieve meaningful improvement. In human medicine, compression bandaging is commonly used for lymphedema management [21], but this technique was impractical and poorly tolerated in cats. Ultimately, the edema gradually worsened, the affected skin developed erythema and ecchymosis, and hemorrhagic effusion accumulated in the thoracic cavity, leading to death despite intensive care. Coagulopathy is a recognized feature in human GLA and CCLA patients [5], and a similar coagulation abnormality may have contributed to the bleeding tendency in this case, although hematologic testing was not performed.

Recent genetic studies have demonstrated that GLA is commonly associated with mutations in PIK3CA, whereas CCLA has been linked to alterations in EPHB4 and ARAF—genes involved in the PI3K/AKT/mTOR and RAS/MAPK signaling pathways [4,6]. These molecular abnormalities are thought to disrupt normal lymphangiogenesis, leading to aberrant development and impaired maturation of lymphatic vessels. Although genetic analysis was not performed in the present case, future investigations using targeted sequencing of formalin-fixed samples may provide insight into the molecular pathogenesis of lymphatic anomalies in animals. Such studies could also lay the groundwork for molecularly targeted therapies including mTOR and PI3K inhibitors which have demonstrated therapeutic benefit in human patients with inoperable CLAs [4,6].

Overall, this case demonstrates that cats can develop systemic lymphatic disorders analogous to human CLAs, underscoring the importance of recognizing these conditions to avoid misdiagnosis as idiopathic or secondary chylothorax. Incorporating the ISSVA classification helps establish standardized terminology and supports comparative research between veterinary and human medicine, ultimately contributing to improved diagnostic accuracy and the development of innovative therapies for lymphatic disorders.

## 4. Conclusions

This case represents the first reported feline example of a CLA exhibiting overlapping features of GLA and CCLA. The combination of systemic lymphatic proliferation, chyle leakage, and diffuse subcutaneous edema indicates a congenital and systemic defect in lymphatic development and function. CT lymphangiography proved to be an effective diagnostic tool for identifying multifocal lymphatic leakage and assessing disease distribution. Although current veterinary therapies remain largely supportive, insights from human CLA research, including molecular diagnostics and targeted therapies acting on the PI3K/AKT/mTOR and RAS/MAPK pathways, may eventually provide novel treatment options. This case underscores the value of adopting the ISSVA classification for veterinary lymphatic disorders, thereby enabling accurate diagnosis and fostering comparative research that bridges veterinary and human medicine.

## Figures and Tables

**Table 1:** Blood examination findings at initial presentation.

Parameter	Unit	Value	Reference Interval
Hct	%	47.7	30.3–52.3
WBC	/μL	15,900	2870–17,020
Plt	10^3^/μL	665	151–600
ALT	U/L	58	22–84
ALP	U/L	138	0–58
TBil	mg/dL	0.1	0.0–0.5
BUN	mg/dL	22.0	17.6–32.8
Cr	mg/dL	0.7	0.9–2.1
TP	g/dL	6.5	5.7–7.8
Alb	g/dL	2.8	2.3–3.5
Glu	mg/dL	153	71–148
TCho	mg/dL	96	95–259
TG	mg/dL	75	16–130
Na	mEq/L	154	147–156
K	mEq/L	3.6	3.4–4.6
Cl	mEq/L	107	107–120
Ca	mg/dL	8.4	8.8–11.9
IP	mg/dL	9.0	2.6–6.0

Hct, hematocrit; WBC, white blood cell count; Plt, platelet count; ALT, alanine aminotransferase; ALP, alkaline phosphatase; TBil, total bilirubin; BUN, blood urea nitrogen; Cr, creatinine; TP, total protein; Alb, albumin; Glu, glucose; TCho, total cholesterol; TG, triglyceride; Na, sodium; K, potassium; Cl, chloride; Ca, calcium; IP, phosphorus.

**Table 2:** Laboratory analysis of pleural and peritoneal effusion samples.

Parameter	Unit	Pleural Effusion	Peritoneal Effusion
TNCC	/µL	14,630	6930
SG	/μL	1.030	1.028
TP	g/dL	4.0	1.4
Alb	g/dL	1.8	0.3
TCho	mg/dL	117	29
TG	mg/dL	>500	>500

TNCC, total nucleated cell count; SG, specific gravity; TP, total protein; Alb, albumin; TCho, total cholesterol; TG, triglyceride.

## Data Availability

The original contributions presented in this study are included in the article/supplementary material. Further inquiries can be directed to the corresponding author(s).

## Supplementary Materials

The following supporting information can be downloaded at: https://www.mdpi.com/article/10.3390/vetsci12121199/s1, Figure S1: The original image of Figure 4A; Figure S2: The original image of Figure 4B; Figure S3: The original image of Figure 4C; Figure S4: The original image of Figure 4D.
