# Supplementary figures and images for: Complex Lymphatic Anomaly Presenting with Chylothorax, Chylous Ascites, and Generalized Subcutaneous Edema in a Young Cat: Comparative Insights Based on the Human ISSVA Classification
Source: Vet Sci. 2025 Dec 15;12(12):1199. doi: 10.3390/vetsci12121199 (PMC12737564; doi:10.3390/vetsci12121199)

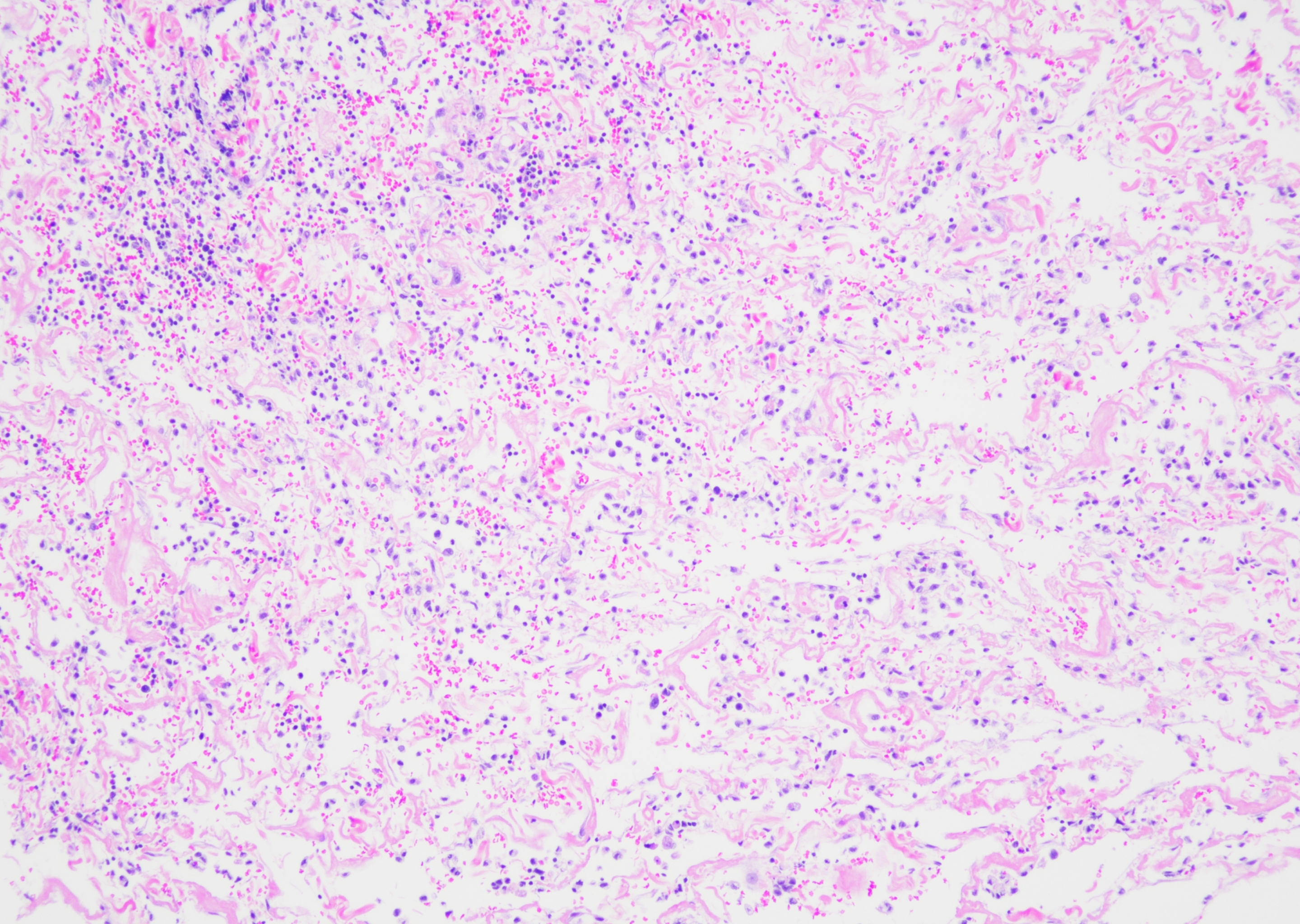

Supplement: Supplementary file 1 [file vetsci-12-01199-s001.zip › Fig4A_original.jpg]

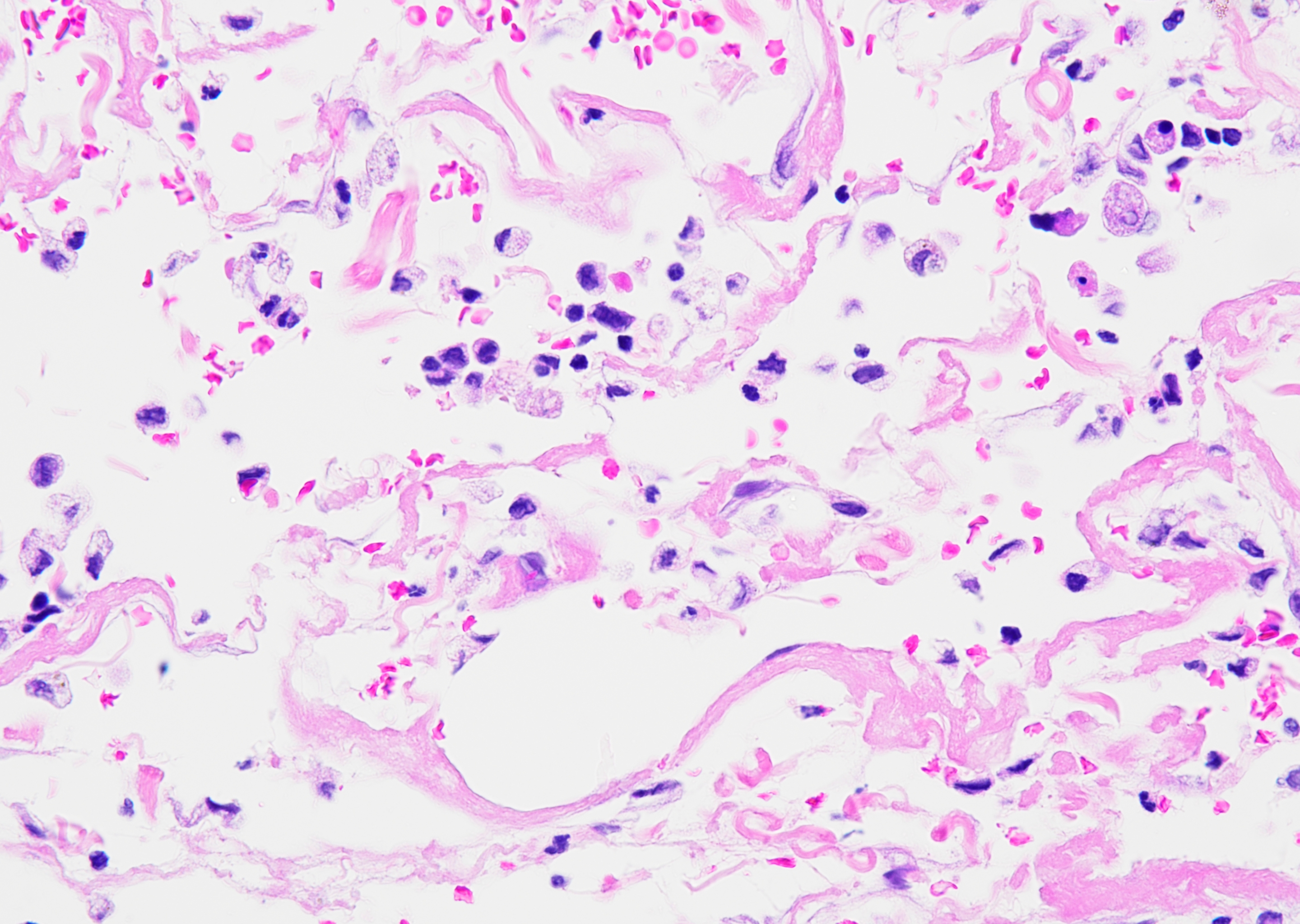

Supplement: Supplementary file 1 [file vetsci-12-01199-s001.zip › Fig4B_original.jpg]

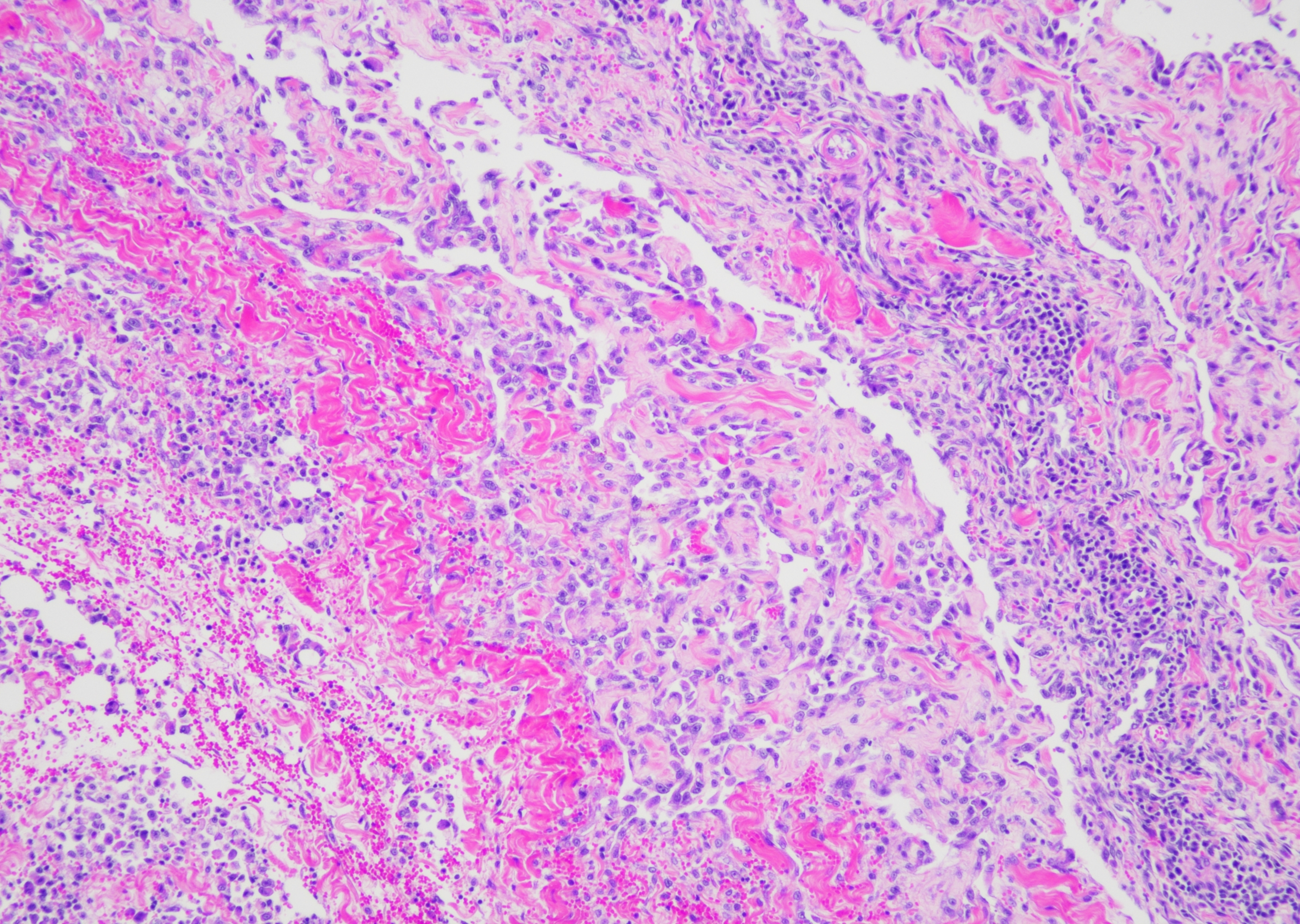

Supplement: Supplementary file 1 [file vetsci-12-01199-s001.zip › Fig4C_original.jpg]

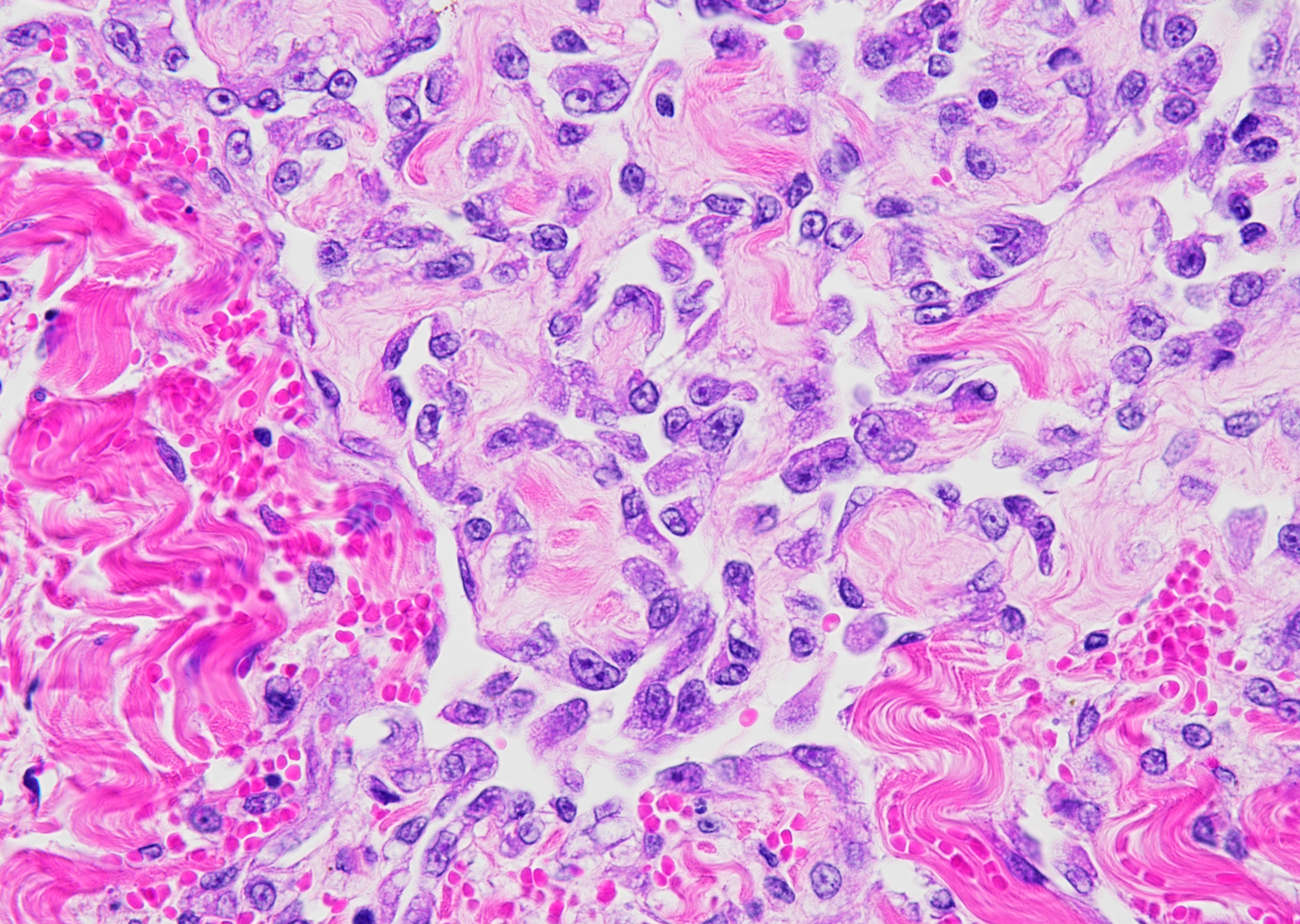

Supplement: Supplementary file 1 [file vetsci-12-01199-s001.zip › Fig4D_original.jpg]
